# Account of a Wound of the Ulnar Artery, at the Wrist, Cured by Tying It up at Some Distance from the Wound

**Published:** 1790

**Authors:** Edward Ford

**Affiliations:** Surgeon of the Westminster General Dispensary


VI. *Account of a Wound of the ulnar Artery, at the Wriſt, cured by tying it up at ſome Diſtance from the Wound. By Mr.* Edward Ford, *Surgeon of the Weſtminſter General Diſpenſary.*

A. B. at No. 11, St. James's Market, applied to me on the 3d of October, 1790, on account of a wound in the wriſt, which he had received a fortnight before by puſhing his hand through a glaſs window.

The wound was on the left ſide, juſt below the carpal ligament, over the os piſiforme. It had bled ſeveral times ſince the accident, and the hæmorrhage had been with difficulty reſtrained by bandages and common dreſſings.

There

There was a pulſating tumour, covered with a thin membrane, where the wound had been received. The pulſation was with difficulty checked by ſtrong compreſſion on the ulnar artery, but returned again on removing the compreſſion.

I dilated the wound inſtantly towards the palm of the hand, and made an attempt to include the veſſel within a ligature by means of the common crooked needle. This proving ineffectual, I repreſented to the patient the neceſſity of a farther dilatation of the wound, in order to reſtrain the hæmorrhage, without injury to the ligaments and tendons of the wriſt; but, notwithſtanding the moſt urgent intreaties, he could not be prevailed on to ſubmit to any farther operation till the enſuing day: he was, therefore, left with a bandage on the wound, and the tourniquet applied on the upper arm, with directions to tighten it if the hæmorrhage ſhould return.

I ſaw him again on the next day, in company with Mr. Lynn, Surgeon of the Weſtminſter Hoſpital, and finding that the bleeding had recurred, it was determined to proſecute the inciſion farther, and to ſecure the artery higher up in the arm.

I began the incifion where the wound had been received, carrying it fuperficially over the carpal ligament, in the direction of the ulnar artery, for fix inches upwards in the arm.

The operation was done with the utmoft caution, to avoid wounding the tendons and mufcles, which were carefully held afide with our fingers as they were expofed by the diffection. This proceeding was of courfe tedious, in order to enfure fafety. The pulfation of the veffel could not be felt; neither could the hæmorrhage be provoked by friction, by putting the arm in warm water, or by flannels applied hot to the part. At length, however, the artery was difcovered, and a ligature carried round it, without including any other part, about an inch and a half above the wrift. Some time was then employed in endeavouring to promote an hæmorrhage from the lower branch of the artery; but this not taking place, the wound was carefully cleaned, united with ftrips of fticking plafter, and rolled up with a flannel roller.

The patient was bled, had an opiate adminiftered, and in every refpect was treated in the moft antiphlogiftic manner.

No circumftance occurred to render it neceffary to open the wound for fix days; no fwelling, ling, tenſion, inflammation, or fever, came on; though it may be remarked that the circular ligament of the wriſt, together with the tendons and muſcles contiguous to the ulna, had been laid bare and expoſed to the air for more than three quarters of an hour during our ſearch for the artery. The ligature came away on the eighth day, and the wound healed rapidly by the firſt intention, ſo that the patient was perfectly well on the 28th of the ſame month.

Though the foregoing operation may have been performed by other ſurgeons, yet as I do not recollect any ſimilar caſe recorded, I flatter myſelf that it is of ſufficient conſequence for inſertion in the London Medical Journal, as it exhibits a certain reſource in a very troubleſome accident, in which other means may have failed.

It is well known that arteries retract very conſiderably in a ſhort ſpace of time after being wounded; and it is not at all times that ſurgical aſſiſtance is to be had at the moment it is wanted.

The ulnar artery is ſo ſituated at the wriſt as to be expoſed continually to accidents; and the riſk of paſſing a ligature deep enough to ſecure the veſſel is ſufficiently obvious to deter any cautious practitioner from uſing the crooked needle needle to any depth in the neighbourhood of ſo many tendons, nerves, and ligaments, as are met with in this part, were he even called upon at the moment of the accident; but when ſome days or weeks may have paſſed over, after the wound has been received, the difficulty muſt be much increaſed, as the artery will probably have retracted.

The method here adopted ſeems leſs exceptionable than that of taking up the veſſel at the wriſt. If the operation is carefully performed, and if inflammation and ſuppuration are prevented by a ſimple treatment of the wound, a proper poſition of the limb, and a cool regimen, it is not likely to deſtroy the uſe of any part of the limb, which might probably happen if a ligature were carried round the artery in the uſual way, ſo as to include any part of the carpal ligament, or any of the nerves or tendons of this part.

In this caſe the patient perfectly recovered, and purſues his occupation as a butcher, having the full uſe of his arm and of his fingers.

*Golden Square,*

October 31, 1790.

